# Nuclear TK1 expression is an independent prognostic factor for survival in pre-malignant and malignant lesions of the cervix

**DOI:** 10.1186/1471-2407-13-249

**Published:** 2013-05-21

**Authors:** Gang Chen, Cheng He, Ling Li, An Lin, Xiongwei Zheng, Ellen He, Sven Skog

**Affiliations:** 1 Department of Pathology, Fujian Provincial Cancer Hospital, Teaching Hospital of Fujian Medical University, Fuzhou, Fujian, 350014, China; 2 Department of Pathology, Fujian Provincial Cancer Hospital, Teaching 472 Hospital of Fujian Medical University, Fuzhou, Fujian, 350014, China; 3 Sino-Swed Molecular Bio-Medicine Research Institute, No. 2–304 Bio-tech Industry Incubator, High-tech Industrial Park, Gaoxin, C. Ave. 1st, Shenzhen, PC. 518057, China

**Keywords:** Cervical lesions, Cervical intraepithelial neoplasia (CIN), Invasive cervical carcinoma, TK1, Ki-67

## Abstract

**Background:**

Thymidine kinase 1 (TK1) is a proliferation biomarker that has been found useful for prognostication in cancer patients. Here we investigate for the first time the use of TK1 expression as a prognostic factor for patients with premalignant and malignant lesions of the uterine cervix.

**Methods:**

TK1 expression was determined by immunohistochemistry in cervical lesions (cervical intraepithelial neoplasia (CIN), n = 216; invasive cervical carcinoma, n = 84). TK1 and Ki-67 expressions and pathological/FIGO stages and age were correlated with 5-year survival by Kaplan-Meier, log rank and COX hazard uni- and multivariate analyses.

**Results:**

TK1 labeling index (LI) was significantly correlated with CIN grades and invasive cervical carcinoma stages, while TK1 labeling intensity was only correlated to CIN grades. TK1 LI was significantly higher compared with Ki-67 LI. TK1 LI correlated significantly to 5-year survival in patients with invasive cervical carcinoma, particularly nuclear TK1 LI. In a multivariate analysis, nuclear TK1 expression was independent prognostic factor in patients with *in situ*/invasive cervical carcinoma or in invasive cervical carcinoma alone. Interestingly, in invasive cervical carcinoma patients with advanced tumors, nuclear TK1 expression could identify patients with significantly better survival rates (80%), while Ki-67 could not.

**Conclusions:**

Nuclear TK1 expression in early grade CIN predicts risk for progression to malignancy. Nuclear TK1 expression is also a prognostic factor for treatment outcome, particularly in patients with advanced cervical carcinomas. Nuclear TK1 expression is more useful than Ki-67 and pathological/FIGO stages.

## Background

Cervical cancer is the third most common malignancy in women and a major cause of morbidity and mortality, particularly in developing countries [1]. However, in industrial countries, the incidence and mortality rates of cervical cancer are decreasing [2]. A Hong Kong based study reported in 2011 an overall 5-year survival rates of 90.9%, 71.0%, 41.7% and 7.8%, respectively in FIGO stages I, II, III, and IV of invasive cervical cancer [2]. Patient age, FIGO stage and histology were independent prognostic factors.

Any grade of cervical intraepithelial neoplasia (CIN) has a potential risk of progression to invasive cervical carcinomas, in particular [3]. Therefore, much research has focused on the eradicating of CIN to prevent development of invasive cancer.

The Pap test smear (cervical cytology smear) has been widely used for detection of CIN and cervical cancer. An human papilloma virus (HPV) intervention trial screening study has shown on HPV DNA testing to permit earlier detection of clinically relevant CIN grade II, which, when adequately treated, should improve protection against progression to CIN grade III or cervical cancer [4,5]. Although HPV infection is regarded as the main etiologic factor for the development of cervical carcinoma, cell proliferation or apoptosis does not correlate with the level of HPV infection [6]. However, the proliferation marker Ki-67 and/or p16 showed correlation with CIN grade I/II and progression-risk to CIN III [7-9]. Automated detection of dual p16/Ki-67 nuclear immune-reactivity in a liquid-based Pap test has been introduced for the analysis of cervical lesions [10].

Recently a cell cycle–dependent marker, thymidine kinase 1 (TK1), has been introduced to evaluate tumor proliferation by immunohistochemistry. A basic study on the expression of TK1 and Ki-67 demonstrated activated G1 cells to show higher TK1 expression compared with Ki-67 [11]. A highly specific TK1 monoclonal antibody developed by our laboratory has been used to assess the proliferation rate of benign, pre-malignant and malignant cells in breast [12] and prostate lesions [13,14]. TK1 was also more sensitive than Ki-67 in cancer patients with prostate [13] and ovary [15] carcinomas. Higher TK1 expression was found in clear cell and papillary renal cell carcinomas (RCC) compared with oncocytomas and normal kidney [16]. Higher expression of TK1 in prostate carcinoma was associated with a shorter interval to recurrence and development of metastasis [14]. Furthermore, TK1 expression was an independent prognostic factor for pathological T1 (pT1) lung adenocarcinoma [17]. In the pT1 patients with stromal invasion grade III, low TK1 level was correlated with good survival. The intensity of TK1 staining was found to be a prognostic factor in RCC patients [18]. Furthermore, the serum TK1 level is a useful marker for prognostication and monitoring of cancer treatment, as well as serving as a potential biomarker for early detection of cancer in health screening setting [19-21].

To date, no studies have been performed on TK1 expression in premalignant and malignant cervical lesions. This study aimed to determine TK1 expression in these lesions and its correlation with outcome. TK1 was found to be a more reliable prognostic marker than Ki-67 and pathological/FIGO stage.

## Methods

### Patients

Specimens were collected from a cohort of 2,840 patients with uterine cervical lesions treated at Fujian Provincial Tumor Hospital, China, from January 2006 to December 2009. Invasive carcinoma account for a high proportion of the cervical lesions because the hospital is an Oncology Center. From this cohort, 300 specimens were randomly selected (CIN I, n = 78; CIN II, n = 64; CIN III, n = 74; invasive cervical squamous cell carcinoma, n = 84). All specimens were formalin-fixed and paraffin-embedded. The histology and diagnosis confirmed on hematoxylin-stained sections. The lesions were classified according to the International Federation of Gynecology and Obstetrics (FIGO) [22] and the International Union Against Cancer (UICC) TNM staging system [23]. The characteristics of the patients are shown in Table 1. In the following text the CIN III/carcinoma in situ/pathological stage 0 group is denoted as “CIN III”. Since the number of the invasive cervical carcinoma patients of pathological stages I was low (n = 3), this group of patients was combined with stage II group (n = 47) for analysis.

**Table 1:** Characteristics of CIN and invasive cervical carcinoma patients

**Type**	**N**
**Age (ys)**	
CIN,	
40.1 (20–80)	216
Invasive cervical carcinoma,	
50.1 (28–81)	84
**Histological type**	
CIN grade I	78
CIN grade II	64
CIN grade III	74
Invasive carcinoma	84
**Pathological stages**	
Cancer in situ, stage 0	74
II	50
III	34
**FIGO stages**	
0	74
IA	1
IB	4
IIA	14
IIB	32
IIIA	2
IIIB	29
IVA	1
IVB	1

The number of CIN patients in the cohort studied was about 20%, which is less than expected. The reason is that this study was performed at an oncology clinic and not at a health center, where the majority of CIN pre-malignancy cases are discovered. Patients with CIN, even CIN III, are not strongly recommended to visit an oncology clinic for further investigation and treatment. However, when the symptoms are obvious, i.e. progress into cervical carcinoma, these patients contact the oncology clinic.

### Study design

The study was to investigate the value of TK1 expression in cervical lesions (CIN and invasive cervical carcinoma) for prognostication. This was performed by determining the LIs of TK1 and Ki-67 in relation to survival. In the first analysis, TK1 expression in tumor cells (total; cytoplasmic; cytoplasmic+nuclear; intensity) was determined in relation to 5-year survival of patients with CIN grade III and invasive cervical carcinoma, as one group. Ki-67 expression and survival rates of the patients of different pathological/FIGO stages were used as controls. In the second analysis, TK1 expression was analyzed in relation to 5-year survival of invasive cervical carcinoma patients with advanced tumors (pathological stage III/FIGO stages IIA – IV). Ki-67 expression and pathological stage IIB/FIGO stage IA, IB and IIA were used as controls. The end-points were the number of deceased patients and the 5-year survival rates. The variables considered were CIN grades, tumor pathological stages, FIGO stages, age, TK1 labeling index (LI) of whole tumor cell, cytoplasmic/nuclear TK1 LI, TK1 intensity and Ki-67 LI. Three hundred specimens were studied to allow for adequate statistical analysis.

### Treatment

Patients treatment was in accordance with recommendations of the FIGO [22]. Briefly, patients with CIN I (n = 78) were checked once per year and were not treated. Patients with CIN II (n = 64) were treated by LEEP (loop electrosurgical excision procedure) procedure or laser surgery, while patients with CIN III (n = 74) were treated by surgery. Patients with invasive cervical carcinoma (n = 84) received surgical treatment combined with chemotherapy/radiotherapy according to the FIGO guidelines [22].

### Follow-up information

In total, 106 patients (CIN III, n = 45; invasive cervical carcinoma pathological stage II (n = 28), III (n = 33) or FIGO stages IA+IIA (n = 13), and IIB+IV (n = 48) were followed-up over 5 years using information obtained from the medical records and telephone contacts. Information was collected from 4^th^ January 2006 to 23^rd^ April 2012. The CIN patients with stages I and II were also followed up for 5 years, but no death were reported, and thus no statistical COX analysis could be performed.

### Immunohistochemical staining

Immunohistochemical staining was carried out using the EnVision System according to the manufacturer’s instructions (Maxin Biotech, Fuzhou, China), as previously described [12,17]. In brief, two serial sections were used for the staining of human TK1 monoclonal antibody (800 × PBS dilution of 1 mg/ml, SSTK Biotech. Ltd., Shenzhen, China) and Ki-67 mAb (MIB-1, 50 mg IgG1/l, Dako, Copenhagen, Denmark), respectively. The anti-TK1 monoclonal antibody has previously been quality controlled and characterized [12]. The quality of the TK1 monoclonal antibody used in this study was also confirmed by independent research groups [14,16,18,21].

**Figure 1:**
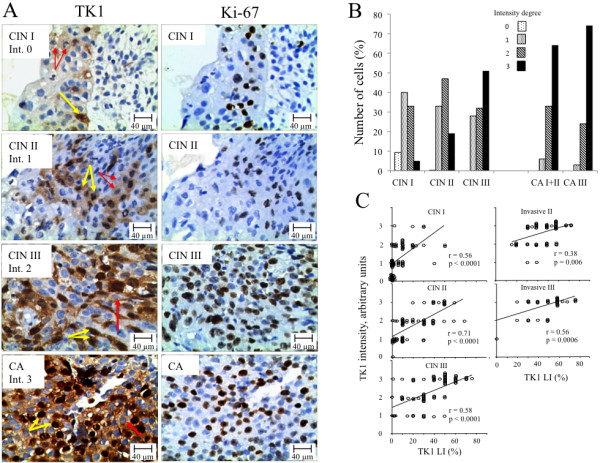
**TK1 and Ki-67 immunohistochemistry staining of CIN and invasive cervical carcinomas. A**) Example of TK1 and Ki-67 immunohistochemistry staining of CIN I, CIN II, CIN III and invasive cervical carcinomas (CA). The intensity (Int.) of the TK1 staining was scored as 0, 1, 2, and 3. Red arrows indicate cells with cytoplasmic TK1 staining only; yellow arrows indicate cells with both cytoplasmic and nuclear TK1 staining. Magnification x 400. **B**) Relative number of CIN and invasive cervical carcinoma patients with various TK1 staining intensities. **C**) Correlation between TK1 LI and TK1 intensity of CIN I to III lesions and of invasive cervical carcinomas with pathological stages II and III. Pearson-correlation statistical values are shown (r = regression coefficient).

At least 100 lesional cells were counted in approximately 10 microscopic fields at a magnification of x 400. The expression of TK1 and Ki-67 were determined by the percentage of stained cells, denoted as labeling index (LI). The LI of TK1 was assessed as “total TK1” (TK1 was expressed in the cytoplasm alone and in both the cytoplasm and nucleus). The “cytoplasmic and nuclear TK1” refers to simultaneous TK1 expression in the cytoplasm and nuclei. The LI of Ki-67 was determined as the percentage of cells with nuclear staining. In addition to LI, the intensities of the staining of TK1 were determined by a semi-quantitative score system described by Gakis et al. [18]. Briefly, the TK1 intensities were checked visually at a magnification of x 160 and divided into four groups denoted 0, 1, 2 and 3. Examples of the TK1 intensities are shown in Figure 1A. The intensity of Ki-67 was also evaluated. However, since there was no change in the intensity of Ki-67 it was not evaluated in detail. One pathologist (Cheng He) determined the expression of TK1 and Ki-67 LI twice and the results were re-checked once by a second pathologist (Gang Chen). The counting was performed blind.

### Statistical analysis

Statistical significance was calculated by two-tailed t-tests (SPSS Statistics V17.0, IBM, USA) and Chi-square test by correlation-Pearson test (Analysis-it, UK). Kaplan–Meier and log-rank tests (SPSS Statistics V17.0, IBM) were used when calculating the statistical significances of the survival rates, while COX regression analysis was used for the uni- and multivariate analysis (SPSS Statistics V17.0, IBM). When p-values were > 0.05 no data was given on the hazard ratios and 95% confidence interval (CI) by the statistical program (SPSS Statistics V17.0, IBM). P-values of < 0.05 were considered statistically significant.

**Figure 2:**
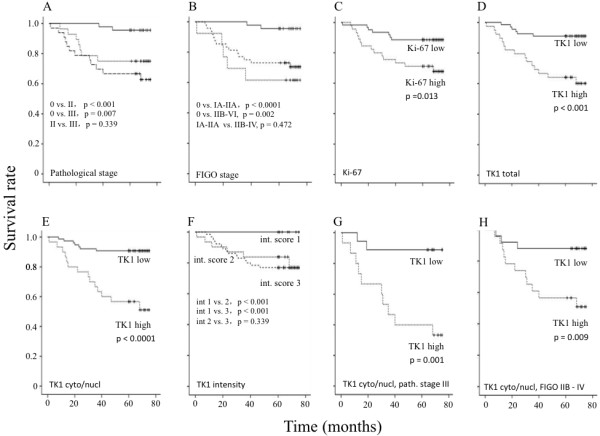
**Five-year survival of CIN III and invasive cervical carcinoma patients in relation to LI.** (**A**) Five-year survival rate of pathological stages 0 to III (0 solid line, II dotted line and III dashed line) and **(B)** FIGO stages 0 to IV (0 solid line, IA to IIA dotted line and IIB to IV dashed line) of CIN III and invasive cervical carcinoma patients combined (n = 106). Five-year survival rate in relation to LI of Ki-67 (**C**), total TK1 (**D**), cytoplasmic/nuclear TK1 (**E**), TK1 intensity (**F**), TK1 cytoplasmic and nuclear of pathological stage III (n = 33) (**G**) and FIGO stage IIB to IV (**H**). Low LI (solid line) and high LI (dotted line). The TK1 intensity (**F**) was scored as 1 (solid line), 2 (dotted line) and 3 (dashed line). Significant log rank values between the survival curves are shown. The solid dots in the survival curves show the times of censored observations.

ROC analysis (Analysis-it, UK) was used when determining the cut-off values of the LI of TK1 and Ki-67. The cut-off value of total TK1 LI was set to 70.0% (ROC-value 0.86, p < 0.0001), cytoplasm and nuclear TK1 to 50.0% (ROC-value 0.81, p < 0.0001) and Ki-67 to 55.0% (ROC-value 0.81, p < 0.0001). Patients with a LI below the cut-off values were denoted as “low” and above the cut-off value as “high” (Figure 2).

The study was approved by the Committee of Research Ethics at Fujian Provincial Cancer Hospital and Teaching Hospital of Fujian Medical University, Fuzhou, Fujian, China. All the patients gave informed consent to participate in this study, which was conducted in accordance with the Helsinki Declaration of 1983.

## Results

### LI of TK1 and Ki-67

The LI of TK1 and Ki-67 are shown in Table 2. While Ki-67 was exclusively found in the nuclei of cells (Figure 1A), TK1 was expressed in both the cytoplasm and nuclei, or in the cytoplasm only (Figure 1A). There were no cells with only TK1 in the nuclei (Figure 1A).

**Table 2:** Labeling index (LI) in relation to grades of CIN and stages of invasive cervical carcinoma

**Type**	**Grade/stage**	**No**	TK1 total*	**P**	TK1 cyto + nucl**	**P**	**Ki-67**	**P**
CIN	Grade I	78	17.5±16.7		7.3±11.2		10.8±13.0	
				<0.0001		<0.0001		<0.0001
	Grade II	64	37.2±22.9		22.1±18.7		26.5±21.2	
				<0.0001		<0.0001		<0.0001
*Carcinoma in situ*	Grade III	74	54.6±20.5		36.6±18.9		43.9±23.1	
(CIN III)								
				0.001		0.041		<0.0001
Invasive CA	Stage II	50	66.6±17.8		43.5±18.4		60.5±23.1	
				0.029		0.117		0.701
	Stage III	34	74.9±15.4		49.7±18.4		62.5±23.2	

*TK1 expression in cytoplasm alone + TK1 expression in both cytoplasm and nuclear; ** TK1 expression in cytoplasm + nuclear; No = number of patients. CA = carcinoma.

For CIN lesions, the LI of total TK1, cytoplasmic and nuclear TK1, and Ki-67 increased significantly from CIN grade I to grade III (Table 2). In patients with invasive cervical carcinoma, only the LI of total TK1 increased significantly, compared with CIN III (Table 2). The LI of TK1 was significantly higher compared with the LI of Ki-67 (p > 0.05). The ratio between the TK1 expression in the cytoplasmic and nuclear group and the total TK1 group increased from CIN grade I to grade III (I = 0.42, II= 0.61, III= 0.67), but did not increase further in the invasive carcinoma pathological stages II and III (II = 0.63, III = 0.66).

### Intensity of TK1 and Ki-67 expression

The intensity of TK1 expression increased significantly from CIN I to CIN III and from CIN III to the invasive cervical carcinoma pathological stage II/FIGO stage IA + IIA, but did not increase further in the advanced tumor stages (Table 3). There were no changes in the intensity of the Ki-67 staining among CIN grades and stages of the invasive cervical carcinoma (Figure 1A). However, there was a correlation between changes of TK1 LI and TK1 intensities in both CIN and invasive cervical carcinoma patients (Figure 1C).

**Table 3:** Number of patients with low (score 0 and 1) or high (score 2 and 3) TK1 intensity in relation to CIN and invasive cervical carcinoma (CA)

**Type**	**Grade/stage**	**Low**	**High**	**P**
CIN	Grade I	48	30	
				0.024
	Grade II	22	42	
				0.012
Cancer *in situ*	Grade III	12	62	
				0.026
Invasive CA	Stage II	2	48	
				0.83
	Stage III	1	33	

### Survival of patients in relation to pathologic and FIGO stages

Of the patients with CIN III, and pathological stages II and III cervical carcinoma, 19.8% (21/106) died during the 5 year follow-up period, of which only two patients with CIN III died. Both of these patients showed a total TK1 LI of 80% and cytoplasmic/nuclear TK1 LI of 60%. The TK1 intensity score was 3. The LI of Ki-67 were 83% and 3%, respectively. One of the patients died at 31 months and the other at 47 months.

The 5-year survival rate of the CIN III patients was 95.6%, while that of patients with pathological stages II and III were 75.0% and 64.7%, respectively. Kaplan–Meier survival curves and log-rank test showed that the survival of the CIN III patients was significantly better compared to the invasive cervical carcinoma patients with pathological stage II (X^2^ = 12.5, p < 0.0001) and III (X^2^ = 7.3, p = 0.007) (Figure 2A). There was no significantly difference in the survival between pathological stage II and III of the invasive cervical carcinoma patients (X^2^ = 0.9, p = 0.339) (Figure 2A). Using the FIGO classification system, the 5-year survival rate was 100% at FIGO 0, 69.2% at FIGO IA+IIA and 64.5% at FIGO IIB+IV (Figure 2B). Kaplan-Meier survival curves and log rank tests showed a significant difference in the survival rate for FIGO stage 0 patients versus FIGO IA+IIA (X^2^ = 13.2, p < 0.0001) or versus IIB+IV (X^2^ = 9.5, p = 0.002) (Figure 2B). No significant difference was found between patients with FIGO IA+IIA versus IIB+IV (X^2^ = 0.5, p = 0.472) (Figure 2B).

### TK1 and Ki-67 in relation to survival

#### Death of CIN III and invasive cervical carcinoma patients

The percentage of deceased patients in the low and high total TK1 LI was 9.0% and 38.5%, respectively (X^2^ = 8.6, p = 0.003), while the corresponding values for the cytoplasmic/nuclear TK1 LI was 9.2% and 46.7%, respectively (X^2^ = 11.4, p < 0.001). The percentages of deceased patients with low and high Ki-67 LI groups were 12% and 31%, respectively (X^2^ = 4.1, p = 0.042).

#### Survival of CIN III and invasive cervical carcinoma patients

Kaplan-Meier 5-year survival curves and log rank analyses showed significant differences in the survival rates between low and high TK1 LI patients in the total TK1 group (Figure 2D), cytoplasmic/nuclear TK1 group (Figure 2E), and TK1 intensity groups (Figure 2F), and Ki-67 (Figure 2C) group (Tab. 4). The significant value of the cytoplasmic/nuclear TK1 group was higher compared with the total TK1 group (Tab. 4, Figure 2D and E).

#### Survival of invasive cervical carcinoma patients

There was a significant difference in the survival of patients with low and high TK1 LI in the cytoplasmic/nuclear TK1 group, and also in the total TK1 group, but not in the Ki-67 group (Table 4). The survival curves were very similar for those of the CIN III and invasive cervical carcinoma (see Figure 2), and thus we show the survival curves of invasive cervical carcinoma patients alone.

**Table 4:** Log rank and Cox multivariate analysis of Kaplan-Mayer survival curves of CIN III and invasive cervical carcinomas patients

		**Log rank**			**Multivariate**	
**Variable**	**X**^**2**^	**DF**	**P**	**Hazard risk**	**95% CI**	**P**
**CIN III + invasive CA (n=106)**						
"Total TK1"	8.60	1	0.003	3.30	1.3 – 8.8	0.015
"Cyto+nucl TK1"	11.39	1	<0.001	4.30	1.7 – 10.9	0.002
Ki-67	4.13	1	0.042	nd	nd	0.541
Pathological stages:						
0 versus II	12.51	1	<0.001	6.53	1.35-31.38	0.020
0 versus III	7.315	1	0,007	nd	nd	0.065
FIGO stages:						
0 versus IA-IIA	13.23	1	<0.001	9.19	1.68-50.35	0.011
0 versus IIB-IV	9.47	1	0.002	nd	nd	0.060
Age	0.25	1	0.873	nd	nd	0.160
**Invasive CA (n=61)**						
"Total TK1"	3.51	1	0,061	nd	nd	0.060
"Cyto+nucl TK1"	6.59	1	0,01	7.19	1.7– 29.7	0.015
Ki-67	0.00	1	0.999	nd	nd	0.468
FIGO stages IA+IIA versus IIB+IV	0.44	1	0.509	nd	nd	0.889
Pathological stage II versus III	0.91	1	0.329	nd	nd	0.280
Age	2.27	1	0.132	nd	nd	0.180

CA = carcinoma ; cyto = cytoplasma; nucl = nuclear; df = degree of freedom; nd = no data.

#### Survival of high-risk invasive carcinoma patients

There was a significant difference in the survival of advanced-staged patients between the low and high TK1 LI patients groups (pathological stage III, X^2^ = 10.25, p = 0.001, n = 33; FIGO stages IIB – IV, X^2^ = 8.78, p = 0.009, n = 48) (Figure 2G, 2H). The survival rate of the low TK1 LI group was about 80%, compared with about 40% for the patients with high TK1 LI. There was also a significant difference in the survival for total TK LI, at least in the FIGO classification, between low and high TK1 LI (pathological stage III, X^2^ = 2.65, p = 0.102, FIGO stages IIB – IV, X^2^ = 4.19, p = 0.041). No such differences were found between low and high Ki-67 LI groups (pathological stage III, X^2^ = 0.196, p = 0.658; FIGO stages IIB – IV, X^2^ = 1.75 p = 0.186). The percentage of patients with better survival in the advanced tumor group was 54.5%, based on the cytoplasmic/nuclear TK1 LI. On the contrary, in the low risk group of invasive carcinoma, there was no difference in the survival between the low and high TK1 LI groups (cytoplasmic/nuclear LI) (pathological stage II, X^2^ = 0.047, p = 0.828; FIGO stages IA- IIA, X^2^ = 1.514, p = 0.219) or in the low and high Ki-67 LI groups (pathological stage, X^2^ = 0.093, p = 0.760; FIGO stages IA-IIA, X^2^ = 0.028, p = 0.866). These results suggest that it is possible to identify patients in the advanced tumor group that still have a good prognosis.

### COX uni- and multivariate analyses

As no CIN I and CIN II patients died, survival and COX uni- and multivariate analyses could not be performed on this group of patients. The prognostic factors that showed significance in the univariate COX analysis were further tested by the multivariate COX analysis. The COX uni- and multivariate analyses were performed on all patients with cervical carcinoma, i.e. CIN III (carcinoma in situ, n=45) and invasive cervical carcinoma (n =61) as one group, or on invasive cervical carcinoma alone (n =61) (Table 4). The results from the univariate analysis are not shown.

Of the variable prognostic factors studied (pathological stages, FIGO stages, TK1 [TK1 total expression, cytoplasmic/nuclear expression], Ki-67 and age), CIN III versus pathological stage II, FIGO stages 0 versus IA – IIA, total TK1 and cytoplasmic/nuclear TK1 type of expression were found to be independent prognostic factors (Table 4). In a corresponding COX analysis of the invasive cervical carcinoma patients alone (pathological stages II and III, n = 61, FIGO stages IA+IIA and IIB+IV, n = 61), only cytoplasmic and nuclear TK1 expression was found to be an independent prognostic factor for survival (Table 4).

## Discussion

In this study investigating on the expression of TK1 in pre-malignant and malignant neoplasms of the cervix, TK1 LI was found to be a more reliable prognostic marker for 5-year survival than pathological stages, FIGO stages and Ki-67, as demonstrated by LI and 5-year survival data. COX multivariate hazard analysis also shows that the expression of TK1 in the nuclei of tumor cells is a markedly independent prognostic factor for both CIN III and invasive cervical carcinoma patients, while Ki-67 was not. Thus, nuclear TK1 expression is a reliable prognostic factor in CIN patients, a group of cervical lesion patients that respond positively to treatment. Furthermore, since nuclear TK1 expression is correlated with advanced stage of invasive cervical carcinomas (pathological stage III/FIGO stage IIA – IV), a low TK1 LI can help to identify with a better survival. Thus, the low TK1 expression in the tumors in these patients might indicate that these tumors have a lower proliferation rate, and may be advantageous for patient survival. Further studies may help determine if less aggressive therapy may be tried to reduce treatment morbidities for these patients.

TK1 is a key kinase in the one-step salvage pathway by which thymidine is introduced into DNA via the salvage pathway [19]. Thus, TK1 participates in DNA synthesis and is therefore closely related to the S-phase of the cell cycle, and is correlated with proliferation [18-21,24]. An important observation in this study is that the TK1 intensity (TK1 synthesis rate) increases from CIN grade I to CIN grade III, but does not further increase in invasive cervical carcinomas. Thus, TK1 intensity seems to be a prognostic factor particularly when pre-malignant cervical lesions progress to malignancy. Although the intensity of TK1 expression is not an independent prognostic factor, it may be of benefit to use both the LI of TK1 and the intensity of the TK1 expression when judging the prognosis of pre-malignant patients, particular in the earlier stages of CIN patients (CIN I and CIN II). The significant correlation between TK1 LI and TK1 intensity shows that higher numbers of proliferating tumor cells are linked to an elevated synthesis rate of TK1. TK1 intensity has similarly been found in other studies to be a prognostic factor in RCC patients [18].

TK1 expression was originally found in the cytoplasm of cells growing *in vitro* (cell lines) [12,24]. TK1 expression was, subsequently, also found in nuclei of different types of tumor tissues [12 – 18]. The reason why TK1 is expressed in the nucleus is still debated, but it has been suggested that this may be result from an elevated concentration of TK1 in the cytoplasm. It has also been suggested that nuclear TK1 is involved in the repair of DNA [25]. In this study, the ratio of nuclear TK1 to cytoplasmic TK1 increases from CIN grade I to CIN grade III, indicating that nuclear TK1 is not due to increasing concentration in the cytoplasm, but to an independent event, for example DNA repair. This is supported by the fact that the survival of patients with high nuclear TK1 expression is significantly less compared to patients with low nuclear TK1 expression and high cytoplasmic expression. Efficient repair of damaged DNA, caused by the chemo and/or radiation therapy, may enhance the survival of tumor cells, but reduce patient survival. In addition, the expression of TK1 in the nucleus is the strongest independent prognostic factor in this study.

In this study two patients with CIN III died. Both patients showed high total TK1 LI (80%) and high cytoplasmic/nuclear TK1 LI (60%) and also a high score of intensity TK1 (score 3). Ki-67 only gave a high LI value in one of the patients and there was no difference in the Ki-67 intensity. Further studies of CIN III may help determine if nuclear TK1 expression is helpful for clinical decision regarding the treatment of individual patients with CIN III. Early discovery of pre-malignancy combined with appropriate treatment may promote a better outcome.

The Pap smear and HPV DNA test can reveal abnormal epithelial cells or presence of high-risk HPV, but these tests do not assess the proliferation rate of cells, which is an important factor for the development of cancer in later life. Nuclear TK1 expression in patients with CIN grade I to III can provide reliable proliferation rate information that is useful for early risk assessment of cancer progression and treatment choices for individuals.

This is a pure immunohistochemical study without molecular work-up, which may limited the understanding of the biological aspects of the findings. Nonetheless, the results do suggest possible application of the findings in clinical management of patients with CIN and cervical cancer. In addition, the possibility to identify patients with better survival by “just” TK1 immunohistochemistry shows the potential of immunostaining techniques alone.

## Conclusion

Nuclear TK1 expression in tumor cells of cervical lesions is an independent prognostic factor, and is important for the judgment of the prognosis of CIN patients, and invasive cervical carcinoma patients. Nuclear TK1 expression is associated with aggressive features of cancer, as demonstrated by its prognostic significance in terms of 5-year survival rates.

## Competing interests

GC, CH, LL, AL and XZ have no competing interest. EH and SS are owner of Biomedical Scandinavia AB.

## Authors’ contributions

GC, CH, LL, AL and XZ made substantial contributions in the collection, analysis and interpretation of data. EH and SS were responsible for analysis of the data and writing the manuscript. GC gave the final approval of the final version to be published. All authors read and approved the final manuscript.

## Pre-publication history

The pre-publication history for this paper can be accessed here:

http://www.biomedcentral.com/1471-2407/13/249/prepub
